# Obesity-related abnormal eating behaviors in Type 2 diabetic patients

**Published:** 2013

**Authors:** Aydan Ercan, Gul Kiziltan

**Affiliations:** 1 Aydan Ercan, Baskent University, Health Science Faculty, Department of Nutrition and Dietetics, Ankara, Turkey.; 2 Gul Kiziltan, Baskent University, Health Science Faculty, Department of Nutrition and Dietetics, Ankara, Turkey.

**Keywords:** Binge eating, Eating behaviors, Night eating, Obesity, Type 2 diabetes mellitus

## Abstract

***Objectives:*** To determine the obesity-related abnormal eating behaviors in Type 2 diabetic patients.

***Methodology:*** It was a cross-sectional study involving 120 Type 2 diabetic patients. Body weight and height of the individuals were measured and body mass index (BMI) was calculated. Bulimic Investigatory Test-Edinburg (BITE) was used to measure binge eating attitudes. Night eating symptoms were defined as consuming >25% of daily energy after suppertime. To determine the glycaemic control, HbA1c levels were analyzed. Data analyzed by SPSS 13.0 for Windows.

***Results: ***The mean age of the patients was 55.3±9.29 years. The mean diabetes duration was 11.1±2.04 years. The 50% of the patients had a BMI higher than 25kg/m^2^. The obese diabetic patients were more likely to have diabetic complications than non-obese subjects (*x*^2^ = 8.588, p=0.040). The percentages of the diabetic patients who met the criteria for binge eating and night eating were 17.2% and 42% respectively. Half of the patients were skipping a main meal per day. The frequencies of obesity-related abnormal eating behaviors were not statistically different in obese patients versus non-obese participants (p>0.05). The mean HbA1c levels were higher in diabetic patients than biochemical references and there was a significant difference between obese and non-obese patients (p<0.05).

***Conclusions: ***Obesity-related abnormal eating behaviors were prevalent in Type 2 diabetic patients and related with diabetic complications and glycaemic control.

## INTRODUCTION

Prevalence of diabetes in adults worldwide was estimated to be 4.0% in 1995 and to rise to 5.4% by the year 2025. It is higher in developed than in developing countries. The number of adults with diabetes in the world will rise from 135 million in 1995 to 300 million in the year 2025.^1^ Also in Turkey, fast developing country, changing in lifestyle such as urbanization and socioeconomic factors, diabetes and impaired glucose tolerance have become more common and similar with Western countries. In the Turkish Diabetes Epidemiology Study (TURDEP), which was the first and the largest population-based survey, the prevalence of diabetes was 7.2% and of IGT was 6.7%. According to TURDEP data, diabetes is more common in women than in man and the frequencies of diabetes in urban and rural areas were similar. Also from the same data, the prevalence rate of obesity was 22%.^2^

Obesity is a common and ubiquitous disease recently recognized as a major health problem in all age groups and is quite a heterogeneous condition not only for the different related medical issues but also for the associated psychological and psychiatric conditions. Epidemiological studies have shown that obesity is an important risk factor for the development of Type 2 diabetes mellitus. It was reported that, about 80% of all Type 2 diabetics are overweight.^3^ Besides obesity, abnormal eating and eating disorders are major public health problems. The disturbed eating patterns associated with such eating disorders can have significant negative consequences in diabetic patients, including poorer dietary and glucose control and a greater likelihood of diabetes complications.^4^

Few studies have been addressed to the assessment of eating attitudes and behaviors in patients with Type 2 diabetes.^5^^,^^6^ The prevalence of eating disorders and diabetes mellitus has been studied exclusively in adolescent insulin dependent diabetes mellitus (IDDM) patients. However, only 10% of all diabetics suffer from IDDM.^7^ Studies with non-insulin dependent diabetes mellitus (NIDDM) patients are rare and have been performed only in a small number of patients.^8^^,^^9^

Diabetes mellitus is a disorder that inevitably focuses attention on body weight and diet. Because conflicts may arise concerning autonomy and dependence, a reduction of self-esteem, and stress within the family, it is perhaps not surprising that diabetes is associated with an increased incidence of eating disorders. Constant dieting might cause binging by promoting the adoption of a cognitively regulated eating style, which is necessary if the physiological defense of body weight is to be overcome. By substituting physiological regulatory controls with cognitive controls, dieting makes the dieter vulnerable to disinhibation and subsequently, overeating. According to this theory, dieting and binge eating, for example are closely related and may explain a possible higher prevalence of bulimia nervosa in IDDM and of binge eating disorder (BED) in NIDDM patients.^10^

Binge eating disorder is characterized by a pattern of recurrent eating binges without purging behavior. The population prevalence of BED appears to range from 1% to 2%, although rates in obesity treatment programs are much higher. Relatively little is known about relationship between BED and Type 2 diabetes mellitus.^11^

A less studied, but potentially clinically significant and prevalent form of eating disorder, among diabetic patients is an eating pattern called night-eating syndrome (NES) which is another obesity-related eating behaviors. NES is currently emerging as a potential candidate for a diagnostic eating disorder. Although the definition of NES varies somewhat with investigators and even with the same investigators over time, the basic criteria of NES encompass skipping breakfast, consuming most food in the late evening and at night, and difficulty with falling or staying asleep.^12^

The aim of this study was to determine the percentage of obesity-related eating behaviors- binge eating and night eating- in Type 2 diabetic patients.

## METHODS

***Participants:*** The subjects of this study were recruited from Baskent University Hospital. Within six months a total of 180 eligible patients agreed to participate in this study. From these a total of 52 (28.8%) failed to complete the study so that 128 (73 women, 55 men) completed the survey. Patients with a medical illness unrelated diabetes was excluded from the study. Diabetes duration of the patients’ was at least one year. The study protocol was approved by the Ethical Committee of the University of Baskent. All participating patients gave informed written consent.

**Questionnaire:** *Demographic data obtained from a brief sociodemographic questionnaire included age, gender, education and marital status. Also, a brief structured interview was conducted with all subjects to assess their nutritional habits, diabetes age, diabetes-specific complaints, and current medical treatment. The mean age of the patients was 55.39.29*

 years.

**Body Mass Index:** *Height was measured to the nearest 0.1 cm, and weight to nearest 0.5 kg in light clothing and without shoes. Body mass index (BMI) was calculated by dividing weight in kilograms by the square of height in meters. The World Health Organization (WHO) classification of BMI categories was used to segregate the patients.*^13^ *The patients were grouped into two categories, normal-weight and obese in accordance with the cut-off points of <25 and ≥25.*

***HbA1c measurement:*** Glycaemic control (HbA1c) was determined by high-performance liquid chromatography (HPLC). 

***Nephropathy:*** Diabetic nephropathy has been classically defined by the presence of proteinuria >0.5 g/24 h.

***Retinopathy:*** To detect diabetic retinopathy, the retinal photograph grading was performed. Small changes in the vessels, called microaneurysms and hemorrhages, showed the onset of retinopathy.

***Neuropathy:*** Diagnosing diabetic neuropathy neurological examination was taken. A neurological exam evaluates the nervous system and such functions as reflexes, sensation, movement, balance, coordination, vision, and hearing. Also an electromyography (EMG) tests were used which tests the nerve and electrical activity of muscles.

***Hypertension:*** Hypertension was defined as systolic blood pressure (SBP) ≥140 mm Hg or diastolic blood pressure (DBP) ≥90 mm Hg (average of two readings taken 5 minutes apart). 

*Cardiovascular Diseases: *Diabetic cardiomyopathy was evaluated by noninvasive tests such as blood pressure measurements and EKG (Electrocardiogram).

***Bulimic Investigatory Test, Edinburgh (BITE):*** The Bulimic Investigatory Test, Edinburgh (BITE) proposed by Henderson and Freeman.^14^ The BITE is a 33-item self-report measure, designed to identify subjects with symptoms of bulimia or binge eating. The BITE consists of two subscales: Symptom Scale, which measures the degree of symptoms present, and the Severity Scale which provides an index of the severity of binging and purging behaviors as defined by their frequency. The maximum possible score is 30 for Symptom Scale. A symptom score of 20 or more indicates a highly disordered eating pattern and the presence of binge eating; the medium range (10-19) suggests an unusual eating pattern; the low range (0-10) falls within normal limits. The reliability of BITE was also determined by a pilot study on 50 diabetic patients. The internal consistency (Cronbach’s alpha) of BITE was 0.68 and its interclass correlation coefficient was 0.95 in the pilot study.

***Night-eating symptoms:*** To determine the amount of daily food intake that patients consumed after suppertime, three intern dietitians collected the dietary data using an open ended, interview-administered dietary history. All participants completed detailed three-day food records. Participants were required to maintain the food records for two weekdays, and one weekend day. Prior to completing the food records, all participants were required to attend a one-hour class of verbal instructions on maintaining the food records. Daily food intakes were stratified into 25% increments. We classified individuals stating that they consumed >25% of their daily intake after suppertime as having night-eating symptoms which was first used by O’Reardon et al.^15^

***Statistical analysis:*** Statistical analyses were conducted using the statistical package for the social sciences (version 13.0, SPSS, Chicago). Categorical data were analyzed using Pearson’s chi-squared statistic. Independent sample t-test analyses were used to detect differences between two groups. Values were considered to be significantly different if p<0.05.

## RESULTS

In Table-I, the clinical characteristics of Type 2 diabetes patients are shown. The 50% of the all diabetic patients were obese with a mean body mass index of 28.8±94.47kg/m^2^. The mean diabetes age was 50.3±12.45 years and the mean duration of diabetes was 11.1±2.04 years. The obese patients’ diabetes age was statistically lower (p<0.05) and diabetes age was statistically higher than non-obese patients (p<0.05). The mean serum HbA1c level was 7.4±1.75% (p<0.05). All the patients who participated in this study were diabetics since at least one year as mentioned in the methodology. So, all were under a diet consultation for diabetes management. As we all know that diabetic diets also consist of the weight management issues, so the obesity is a risk factor for these patients in terms of high serum HbA1c levels.

There were statistically significant differences between obese and non-obese subjects. In obese Type 2 patients, the frequency of diabetes complications was higher than the non-obese patients, and this difference was statistically important ( ^2^ = 8.588 p=0.040).

**Table-I:** Clinical characteristics of the Type 2 diabetic patients

	*Obese Type 2 patients * *(n=64)*		*Non-obese Type 2 patients * * (n=64)*	
	*n*	*%*	*n*	*%*
Diabetes complications Retinopathy Nephropathy Hypertension Retinopathy+nephropathy Diabetic foot Cardiovascular disease	5 12 30 4 1 46	7.8 18.7 46.8 6.3 1.5 71.8	4 7 22 3 - 28	6.2 10.9 34.4 4.7 - 43.7
^2^ = 8.588 p=0.040*				
Diabetes age (years) [x ± SD ]*	47.9±14.89		53.8±11.97	
Diabetes duration (years)* [x ± SD ]*	12.4±2.45		10.4±23.56	
HbA1c (%) [x ± SD ]*	8.1±2.14		7.2±1.55	
BMI (kg/m^2^) [x ± SD ]*	30.4±3.83		23.5±0.95	

* p<0.05

Of the 128 patients 22 (17.2%) met the criteria for binge eating. The percentage of obese subjects diagnosed with binge eating was higher than non-obese subjects, but the difference was not statistically significant. The frequency of night eating was 50% in obese and 35.9% in non-obese patients (p>0.05). Obese patients skipped a main meal more than normal patients (54.6% and 46.8% respectively), but there was no statistically significant differences between groups (p>0.05) (Table-II).

**Table-II:** Obesity-related eating behaviors of the Type 2 diabetic patients

	**Obese Type 2 patients ** **(n=64)**		**Non-obese Type 2 patients ** ** (n=64)**	
	**n**	**%**	**n**	**%**
Binge eating (BITE-Edinburg)				
>20 <20	13 51	20.3 79.7	9 55	14.1 85.9
^2^* = 0.470 p=0.493*				
Night eating				
>25% Total Energy <25% Total Energy	32 32	50 50	23 41	35.9 64.1
^2^* = 1.602 p=0.206*				
Skipping main meals				
Breakfast Lunch Dinner	6 21 8	9.4 32.8 12.5	14 11 5	21.9 17.2 7.8
^2^* = 4.503 p=0.212*				

## DISCUSSION

The management of diabetes and its associated health-risk factors are often complex and require considerable patient education and frequent medical monitoring.^16^ The participation of the patients is basic in order to obtain a correct degree of metabolic control; however, this carries a consequence considerable amount of stress.

The constant stress of maintaining tight glycaemic control can result in two types of psychological distress; subclinical emotional distress and diagnosable psychological disorders.^17^ Diabetic patients initially experience high levels of depression and anxiety.^18^ In our study we did not use any psychological tests, it is one of the strenght of this study.

The risk of eating disturbances has been postulated to be higher in diabetic patients than in the general population due to multiple interacting factors related to diabetes and its treatment.^19^ Diabetes management imposes some degree of perceived dietary restraint, particularly patients who eat according to a predetermined meal plan, rather than in response to internal cues for hunger and satiety.

In this study, we aimed to determine the frequency of obesity-related abnormal eating behaviors, binge eating and night eating, in Type 2 diabetic patients. Binge eating and night eating behaviors are two forms of abnormal eating that most commonly affect overweight and obese persons. ^9^ Most Type 2 diabetic patients are obese, and that obesity is often associated with eating disturbances; in fact, binge eating has been reported to be frequent among Type 2 diabetic patients. It should also be considered that abnormalities of eating behavior can affect considerably body weight and metabolic control in Type 2 diabetes.^20^ The disturbed eating patterns associated with such eating disorders can have significant negative consequences in diabetic patients, including poorer dietary and glucose control and a greater likelihood of diabetes complications.^5^^,^^6^ Few studies have addressed the assessment of eating attitudes and behaviors in patients with Type 2 diabetes.^21^^,^^22^

The relationship between higher weight and disordered eating behaviors (DEB) presents a management dilemma for clinicians, since both dietary restraint and higher weight are clear risk factors for the development of eating disorders (ED) and their negative health consequences. It is well-known that diabetes tend to exhibit increased difficulty in maintaining optimal weight and also are more inclined to be concerned about their weight than their non-diabetic counterparts.^23^

Our findings indicated that 50% of the diabetic patients were obese. In obese diabetic patients, the frequency of the diabetic complications such as the cardiovascular diseases, hypertension, retinopathy, nephropathy, diabetic foot was statistically higher than non-obese Type 2 diabetic patients (p<0.05). Also obese patients had poor glycaemic control than non-obese participants (p<0.05).

Binge eating is characterized by eating an objectively large amount of food with a perceived loss of control in a 2-hour period; it is not followed by compensatory behaviors (i.e., vomiting or laxative abuse). Recently, the binge-eating disorder has been recognized as a prevalent finding among the obese subjects.^24^ BED prevalence is estimated at 2% in the general population^11^, 10% to 20% in obesity clinics.^25^ BED may be a fairly common, co-occurring condition in Type 2 diabetes.^6^^,^^8^^,^^21^^,^^22^ Studies report a wide range of prevalence rates of BED in individuals with Type 2 diabetes, from 2.5% to 25.6%.^26^ In this study the BED frequency was determined as 17%. The frequency was higher in obese Type 2 diabetic patients than non-obese patients (20.3% vs 14.1%).

NES is characterized by a delay in the circadian pattern of eating, such that 25% of the daily total caloric intake occurs after the evening meal and/or there are at least three nocturnal awakenings accompanied by eating per week.^15^ The prevalence of NES is 1.5% in the general population^21^, 9% to 14% in obesity clinics^25^, NES is similar to BED in its relationship to eating disordered attitudes and behaviors and increased psychopathology.^27^

This night eating behavior was linked to lower adherence to diet, exercise, and glucose monitoring and increased depressed mood; it produced a higher relative risk for obesity, HbA1c values 7%, and having two or more diabetes complications.^28^ In a prevalence study of NES based on interviews among obese persons with Type 2 diabetes, the observed rate of 3.8% was lower than expected given rates of at least 9% in other obese samples.^9^ In another study, the prevalence of NES among all types of diabetic patients using the evening hyperphagia criterion of 25%, and this study yielded a prevalence of 9.7%.^29^ According to our findings, 42% of the patients had night eating behavior. Our finding of high frequency of night eating behavior was not surprising, because the non-obese diabetic patient’s body mass index was near to the obesity cut-off points. 

Results from previous studies suggest that eating frequency may be causally associated with body weight and weight changes. According to these studies skipping breakfast was associated with increased risk of obesity.^30^ In this study, half of the patients were skipping a main meal per day. Obese Type 2 patients were skipping meals more than non-obese patients but there were no statistically differences between groups (54% vs 46% respectively, p<0.05).

Limitations of this study include its small sample size and we only used a single item for screening for night-eating behaviors.

## CONCLUSION

In conclusion, our study showed that Type 2 diabetes and obesity were like conjuncted-twins and causes poor glycemic control and complications. The frequency of obesity-related abnormal eating behaviors, such as binge eating and night eating, were very common in Type 2 diabetic patients. So, to achieve more success in nutritional management of the diabetic patients it is necessary to take into consideration these issues, as well. Future studies are necessary in large number of Type 2 diabetic populations.
